# Cardiorenal outcomes of weight loss interventions in people with CKD and type 2 diabetes

**DOI:** 10.1093/ndt/gfaf258

**Published:** 2025-12-04

**Authors:** Thomas J Wilkinson, Jonathan Goldney, Thomas Yates, Joseph Henson, Francesco Zaccardi, Kamlesh Khunti, David Webb, Dimitris Papamargaritis, Melanie J Davies

**Affiliations:** NIHR Leicester Biomedical Research Centre, Leicester Diabetes Centre, Leicester, UK; Leicester Diabetes Centre, Leicester General Hospital, University of Leicester, Leicester, UK; NIHR Leicester Biomedical Research Centre, Leicester Diabetes Centre, Leicester, UK; Leicester Diabetes Centre, Leicester General Hospital, University of Leicester, Leicester, UK; NIHR Leicester Biomedical Research Centre, Leicester Diabetes Centre, Leicester, UK; Leicester Diabetes Centre, Leicester General Hospital, University of Leicester, Leicester, UK; NIHR Leicester Biomedical Research Centre, Leicester Diabetes Centre, Leicester, UK; Leicester Diabetes Centre, Leicester General Hospital, University of Leicester, Leicester, UK; NIHR Leicester Biomedical Research Centre, Leicester Diabetes Centre, Leicester, UK; Leicester Diabetes Centre, Leicester General Hospital, University of Leicester, Leicester, UK; NIHR Leicester Biomedical Research Centre, Leicester Diabetes Centre, Leicester, UK; Leicester Diabetes Centre, Leicester General Hospital, University of Leicester, Leicester, UK; NIHR Applied Research Collaboration East Midlands, Leicester Diabetes Centre, Leicester, UK; Leicester Diabetes Centre, Leicester General Hospital, University of Leicester, Leicester, UK; University of Hospitals Leicester NHS Trust, Leicester, UK; NIHR Leicester Biomedical Research Centre, Leicester Diabetes Centre, Leicester, UK; Leicester Diabetes Centre, Leicester General Hospital, University of Leicester, Leicester, UK; NIHR Applied Research Collaboration East Midlands, Leicester Diabetes Centre, Leicester, UK; NIHR Leicester Biomedical Research Centre, Leicester Diabetes Centre, Leicester, UK; Leicester Diabetes Centre, Leicester General Hospital, University of Leicester, Leicester, UK; NIHR Applied Research Collaboration East Midlands, Leicester Diabetes Centre, Leicester, UK

**Keywords:** bariatric surgery, obesity, semaglutide, tirzepatide, type 2 diabetes

## Abstract

**Background and hypothesis:**

In patients with type 2 diabetes mellitus (T2DM) and chronic kidney disease (CKD), there remains a paucity of data from large, high-quality, real-world studies on the efficacy of weight-lowering interventions, including the glucagon-like peptide-1–based therapies semaglutide and tirzepatide, as well as bariatric surgery (BS). This retrospective cohort study evaluates these interventions on cardiorenal outcomes and mortality using health records from TriNetX US Collaborative Network.

**Methods:**

Three cohorts with T2DM and CKD living with overweight or obesity, prescribed semaglutide or tirzepatide, or with a BS procedure were propensity-score matched with a cohort receiving dipeptidyl peptidase 4 inhibitors (DPP4i, weight-neutral control). We investigated the hazard ratio (HR) of: (i) end-stage renal disease (ESRD); (ii) myocardial infarction (MI); (iii) stroke; and (iv) all-cause mortality.

**Results:**

Cohorts included 17 749 (semaglutide, age 64.1 ± 10.9 years, 57.4% female), 4211 (tirzepatide, 63.7 ± 10.8, 58.1%) and 2603 (BS, 56.1 ± 11.2, 74.1%) patients. Compared with DPP4i, semaglutide reduced the risk of ESRD [HR 0.78 (95% confidence interval 0.71 to 0.85)], while tirzepatide showed a reduction of 42% (0.0.58, 0.45 to 0.75). BS lowered ESRD risk (0.79, 0.67 to 0.92). Semaglutide reduced MI (0.80, 0.72 to 0.88) and stroke (0.85, 0.77 to 0.95) risk, while tirzepatide lowered MI and stroke by 24%. BS was associated with reduced MI (0.45, 0.35 to 0.58) and stroke (0.57, 0.44 to 0.74) risk. All-cause mortality risk was reduced by semaglutide (0.64, 0.59 to 0.70), tirzepatide (0.47, 0.35 to 0.63) and BS (0.68, 0.57 to 0.81).

**Conclusions:**

Using a real-world dataset, semaglutide, tirzepatide and BS—interventions shown to elicit weight loss and improve glycaemic control—have a myriad of benefits on cardio-renal outcomes and mortality, supporting their use as disease-modifying therapy options in T2DM and CKD.

KEY LEARNING POINTS **What was known:**

- Patients with type 2 diabetes (T2DM) and chronic kidney disease (CKD) face heightened risks of cardiovascular and kidney-related complications, further exacerbated by overweight or obesity.
- Weight loss interventions, including bariatric surgery (BS), semaglutide and tirzepatide, may mitigate these risks, but real-world effectiveness data are limited.
- Comparative studies on long-term cardiorenal outcomes for these interventions are needed, particularly for tirzepatide in CKD populations

**This study adds:**

- Renal outcomes: tirzepatide, semaglutide and BS significantly reduced the rates end-stage renal disease (ESRD) in people with T2DM and CKD compared with dipeptidyl peptidase 4 inhibitors (DPP4i), with tirzepatide showing the greatest effect.
- Cardiovascular outcomes: all interventions lowered cardiovascular rates, with BS and tirzepatide demonstrating the most substantial reductions in myocardial infarction and stroke events.
- All-cause mortality: tirzepatide was associated with the largest reduction in all-cause mortality compared with DPP4i, followed by semaglutide and BS, indicating a potential protective effect beyond weight loss.

**Potential impact:**

- Clinical practice: incorporating tirzepatide and semaglutide as key treatment options for T2DM and CKD may reduce ESRD and cardiovascular events, particularly in those with overweight/obesity.
- Policy implications: findings support prioritizing non-surgical, weight-lowering interventions to mitigate cardiorenal risks in high-risk populations, potentially informing treatment guidelines.
- Future research: further investigation into tirzepatide’s dual glucose-dependent insulinotropic polypeptide/glucagon-like peptide-1 receptor activation and its direct kidney-protective mechanisms may refine therapeutic strategies for T2DM and CKD.

## INTRODUCTION

Patients living with concomitant type 2 diabetes mellitus (T2DM) and chronic kidney disease (CKD) are at high risk for kidney failure, cardiovascular events and death [1]. Living with overweight or obesity further contributes to the risk of developing both T2DM and CKD and impacts glycaemic control and kidney function once the diseases develop [2, 3]. These conditions are often collectively referred to as ‘cardiovascular-kidney-metabolic syndrome’ [4]. In these patients, overweight and/or obesity is a modifiable risk factor for premature all-cause and cardiovascular disease (CVD)-related mortality and adverse cardiorenal outcomes such as stroke, end-stage kidney disease (ESRD) or need for kidney replacement therapy (KRT) [2, 3, 5].

Interventions aimed at weight loss are recommended for people with T2DM living with overweight/obesity [6]. The ability of weight loss to ameliorate insulin resistance [7] and slow the progression of kidney damage [2] is well established, as is its capability to reduce concomitant CVD risk factors. Whilst bariatric surgery (BS) can elicit substantial weight loss, lowering the risk of kidney failure [2], improving kidney function [8] and blood glucose [9], only a small proportion of people will undergo this intervention as it is not scalable at a population level [10]. Newer glucose-lowering pharmacotherapies, including glucagon-like peptide-1 (GLP-1)-based therapies, promote large reductions in weight [7]. These therapies include semaglutide [the most effective GLP-1 receptor agonist (RA) for weight loss] as well as tirzepatide, a dual GLP-1/glucose-dependent insulinotropic polypeptide (GIP) RA [11, 12] where weight loss approaches that seen following BS [13].

Randomized controlled trials (RCTs) have confirmed the efficacy of semaglutide, licensed for T2DM and obesity, on improving kidney function, metabolic markers, and cardiovascular morbidity and mortality in people with [14, 15] and without [16, 17] T2DM. For example, the FLOW (Evaluate Renal Function with

Semaglutide Once Weekly) trial found semaglutide reduced the risk of kidney outcomes and death from cardiovascular causes in people with T2DM and CKD [18]. However, although data from RCTs are lacking, observational studies have shown that tirzepatide’s superiority over semaglutide extends beyond that of weight loss, with greater effects observed in reducing glycated haemoglobin (HbA1c) [19], all-cause mortality, adverse cardiovascular events and adverse kidney events in T2DM [20, 21]. Further, in a post-hoc analysis of SURPASS-4, tirzepatide decreased albuminuria and total estimated glomerular filtration rate (eGFR) slopes and nearly halved the risk of a composite kidney endpoint compared with insulin use in adults with T2DM [22]. A recent post-hoc analysis of SURMOUNT-1 and -2 showed that tirzepatide was associated with reduced albuminuria without adverse changes in eGFR [23]. Although a Phase 2 (TREASURE-CKD) trial is currently exploring the use of tirzepatide in people with overweight or obesity and CKD (with and without T2DM), evidence for tirzepatide in people with CKD remains limited [24, 25]. Further dedicated research is necessary to elucidate its renal protective effects fully.

There remains a paucity of data from high-quality and large real-world studies on the efficacy of potent weight-lowering medications, including semaglutide and tirzepatide, as well as BS, on long-term outcomes in people living with T2DM and CKD. In particular, with many clinical trials lacking sufficient power (or being stopped early [18]), the evidence of semaglutide on outcomes, such as ESRD, is unclear [15], and it is currently unknown how effects may differ from other weight-lowering therapies, such as tirzepatide and BS, in people with CKD. With the use of GLP1-RA-based therapies increasing, particularly in people with CKD [26, 27], understanding the safety profiles of these therapies and achieving a more comprehensive appreciation of how different weight loss interventions may affect clinical cardiorenal outcomes in real-world populations is important for clinical decision-making.

Using real-world data, this study aimed to explore the effectiveness of different interventions that lead to clinically important weight loss (the GLP1-RA semaglutide, GLP1-RA/GIP tirzepatide and BS) compared with dipeptidyl peptidase 4 inhibitors (DPP4i), a weight-neutral medication often prescribed to patients with advanced CKD or ESRD [28], on cardiorenal outcomes and mortality in people living with T2DM and CKD.

## MATERIALS AND METHODS

This study follows the Reporting of studies Conducted using Observational Routinely-collected health Data (RECORD) guidelines (Supplementary data, Table S1).

### Study design and data collection

This retrospective cohort study identified a cohort from the US Collaborative Network within the TriNetX federated research database, which currently covers data on >101 million patients [29]. The US Collaborative Network constitutes de-identified, pseudo-anonymized electronic health record data contributed by multiple healthcare organizations (HCOs) across the USA which are harmonized into a central database [29]. The data includes demographics, diagnostic codes, medications, laboratory results and procedures. Data are available from the network to be used in real-time for research purposes, utilizing an in-built analysis software that only returns aggregate results.

### Ethics

All data collection, processing and transmission were performed in compliance with all Data Protection laws applicable to the contributing healthcare organizations, including the US Health Insurance Portability and Accountability Act. No further ethical approval was needed, and as the data are routinely collected and fully anonymized, patient consent is not required.

### Cohorts

We compared outcomes across four cohorts. All cohorts included people with T2DM and CKD (Modification of Diet in Renal Disease–derived eGFR value <75 mL/min/1.73 m^2^ within the last 12 months); 75 mL/min/1.73 m^2^ was chosen to evaluate interventions across a broader spectrum of kidney function, but also reflect the same cut-offs used in numerous clinical trials of glucose-lowering agents (e.g. FLOW [18], DAPA-CKD [30]). All included participants were overweight or obese [body mass index (BMI) >25 kg/m^2^ within the last 12 months], with the first-ever prescription of (i) semaglutide; (ii) tirzepatide; (iii) people with a BS procedure (code listed in Supplementary data, Table S2); or (iv) any DPP4i (control). Similar to other studies [31], DPP4i was chosen as the comparator due to these medications being weight-neutral, and having a neutral effect on the kidneys and cardiovascular outcomes [28, 31].

People were excluded if they had: other renal pathology (codes in Supplementary data, Table S3); type 1 diabetes; BMI <25 kg/m^2^; HbA1c not recorded in the last 12 months; previous use of a GLP-1 RA, DPP4i or tirzepatide; or previous BS.

### Outcomes

We investigated the hazard ratio (HR) of the following cardio-renal outcomes: (i) ESRD, a composite of either the first episode of dialysis or an eGFR <15 mL/min/1.73 m^2^; (ii) myocardial infarction (MI); (iii) stroke; and (iv) all-cause mortality. The clinical codes used to identify these outcomes (excluding eGFR, which was taken from harmonized laboratory results) are presented in Supplementary data, Table S4.

### Confounding factors

From HCO records harmonized on the TriNetX platform, potential confounding factors on outcomes were identified up to 12 months before the index date to be controlled for via propensity-score matching. The index date was defined as the first record of semaglutide, BS, tirzepatide or DPP4i at any HCO in the TriNetX network, with data censored at the occurrence of an outcome, or at the most recent visit to an HCO within the TriNetX network up to date of analysis (22 January 2025). These included demographics: (i) age at time of analysis; (ii) age at index; (iii) sex (male/female); (iv) race (American Indian or Alaska native/Asian/Black or African American/Native Hawaiian or Other Pacific Islander/other race/White/unknown race); (v) biochemistry [eGFR (in categories of 10 mL/min/1.73 m^2^ between 75 and 15 mL/min/1.73 m^2^ and <15 mL/min/1.73 m^2^) and HbA1c (in categories spanning 1%, from 6.5% to 11.5%, <6.5% and >11.5%); (vi) BMI in categories of 5 kg/m^2^ between 25 and 55 kg/m^2^, and >55 kg/m^2^; (vii) blood pressure [systolic blood pressure >160 mmHg (yes/no), diastolic blood pressure >100 mmHg (yes/no)]; (viii) past medical history (yes/no) from the following: hypertensive diseases, ischaemic heart diseases, T2DM with kidney complications, T2DM with neurological complications, T2DM with ophthalmic complications, cerebrovascular diseases, peripheral vascular disease, atherosclerosis, nicotine dependence, alcohol-related disorders; (ix) social factors [persons with potential health hazards related to socioeconomic and psychosocial circumstances (yes/no)]; (x) medications (yes/no): any lipid-lowering therapy, any insulin therapy, any antihypertensive therapy, angiotensin-converting enzyme inhibitors, angiotensin II inhibitors, metformin, sulfonylureas, sodium-glucose co-transporter-2 inhibitors (Supplementary data, Table S5).

### Statistics

To reduce the effect of confounding factors, we used propensity-score matching to generate groups with matched baseline characteristics using the factors listed in the above section. Baseline characteristics at index date for each exposed weight-lowering cohort (semaglutide, BS, tirzepatide) compared with the ‘weight neutral’ [32] comparator cohort (DPP4i) were calculated separately, both before and after propensity-score matching. Continuous variables were reported as mean and standard deviation (SD) and categorical variables as number and percentage. Propensity-score matching utilized 1:1 ‘greedy nearest neighbour’ with a calliper width of 0.1 SD. A univariate Cox proportional-hazards model assessed the relative instantaneous rates of cardiorenal outcomes (ESRD, MI, stroke and all-cause mortality), comparing each exposure cohort to DPP4i individually. The proportional hazard assumption was assessed with Schoenfeld residuals and visually with Kaplan–Meir plots (Supplementary data, Fig. S1). All results were reported as HRs with 95% confidence intervals (CIs). Subgroup analysis was conducted with data stratified by sex (male, female), BMI (25–30, >30 kg/m²), and renal function (eGFR <45, 45–60, >60 mL/min/1.73 m^2^). A sensitivity analysis of those with a follow-up time (>1 year) was also performed. Analyses were performed on the TriNetX platform, which utilizes R survival package, version 3.2–3, to undertake Cox regression modelling. To safeguard protected health information, TriNetX automatically rounds patient counts of 1–9, up to 10. This rounding may affect measures of association for small patient counts. Given the potential for individuals to be recognized, composite outcomes were not calculated. Graphs were created in Stata v18.0 and formatted into figures utilizing Inkscape v1.3.2.

### Role of the funding source

The study’s funder(s) had no role in the study design, data collection, data analysis, data interpretation or manuscript writing.

## RESULTS

### Baseline characteristics of the cohorts after propensity-score matching

After matching, a total of 22 604 patients were included in each cohort for the semaglutide vs DPP4i comparison (mean ± SD age at index 64.1 ± 10.9 years, 57.4% female, eGFR 65.0 ± 17.6, BMI 34.5 ± 7.0 kg/m^2^); 6987 patients in each cohort for the tirzepatide comparison (age at index 63.7 ± 10.8 years, 58.1% female, eGFR 64.9 ± 17.2, BMI 35.0 ± 7.0 kg/m^2^); and 2885 patients in each cohort for the BS comparison (age at index 56.1 ± 11.2 years, 74.1% female, eGFR 69.9 ± 20.4, BMI 42.2 ± 8.7 kg/m^2^). After matching, the cohorts were well balanced; Table 1 shows the baseline characteristics of the cohorts after propensity-score matching.

**Table 1:** Baseline characteristics of the cohorts after propensity-score matching.

		Semaglutide vs DPP4i		BS vs DPP4i		Tirzepatide vs DPP4i	
		Exposure (semaglutide)	Comparator (DPP4i)	Exposure (BS)	Comparator (DPP4i)	Exposure (tirzepatide)	Comparator (DPP4i)
Number		22 604	22 604	2885	2885	6987	6987
Age at index, years		63.9 (10.2)	64.3 (11.5)	56.1 (10.0)	56.0 (12.2)	63.4 (9.9)	63.9 (11.6)
Current age, years		67.3 (10.0)	67.7 (11.0)	63.3 (10.8)	63.4 (12.5)	65.1 (9.71)	65.6 (11.3)
Female, *n* (%)		13 019 (57.5)	12 962 (57.3)	2146 (74.3)	2136 (74.0)	4075 (58.3)	4057 (58.0)
Race, *n* (%)	American Indian or Alaska Native	155 (0.7)	134 (0.6)	17 (0.6)	15 (0.5)	52 (0.7)	37 (0.5)
	Asian	913 (4.0)	951 (4.2)	37 (1.3)	40 (1.4)	205 (2.9)	207 (3.0)
	Black or African American	3688 (16.3)	3738 (16.5)	530 (18.3)	556 (19.2)	911 (13.0)	924 (13.2)
	Native Hawaiian or Other Pacific Islander	190 (0.8)	175 (0.8)	16 (0.6)	16 (0.6)	54 (0.8)	44 (0.6)
	Other	772 (3.4)	805 (3.6)	101 (3.5)	100 (3.5)	222 (3.2)	252 (3.6)
	White	15 781 (69.8)	15 677 (69.3)	2020 (70.0)	1979 (68.5)	5223 (74.7)	5170 (73.9)
	Unknown	1105 (4.9)	1124 (5.0)	164 (5.7)	179 (6.2)	320 (4.6)	353 (5.1)
eGFR, mL/min/1.73 m^2^		64.3 (16.5)	65.6 (18.7)	69.1 (18.8)	70.6 (21.8)	64.0 (16.4)	65.7 (17.9)
eGFR categories, *n* (%)	65–75 mL/min/1.73 m^2^	14 164 (62.6)	14 123 (62.4)	1995 (69.1)	2013 (69.7)	4474 (64.0)	4457 (63.7)
	55–65 mL/min/1.73 m^2^	9951 (44.0)	9946 (44.0)	1321 (45.7)	1332 (46.1)	2949 (42.2)	2930 (41.9)
	45–55 mL/min/1.73 m^2^	5810 (25.7)	5839 (25.8)	664 (23.0)	695 (24.0)	1533 (21.9)	1573 (22.5)
	35–45 mL/min/1.73 m^2^	2780 (12.2)	2828 (12.5)	277 (9.6)	293 (10.1)	701 (10.0)	724 (10.3)
	25–35 mL/min/1.73 m^2^	1019 (4.5)	1039 (4.6)	78 (2.7)	77 (2.7)	262 (3.75)	283 (4.1)
	15–25 mL/min/1.73 m^2^	262 (1.2)	280 (1.2)	18 (0.6)	20 (0.7)	72 (1.0)	89 (1.3)
	<15 mL/min/1.73 m^2^	792 (3.5)	791 (3.5)	173 (6.0)	172 (6.0)	133 (1.9)	126 (1.8)
BMI, kg/m^2^		34.8 (7.1)	34.2 (7.0)	42.4 (8.7)	42.0 (8.6)	35.5 (7.0)	34.4 (6.9)
BMI categories, *n* (%)	<25 kg/m^2^	1113 (4.9)	1190 (5.3)	112 (3.9)	129 (4.5)	238 (3.4)	254 (3.6)
	25–30 kg/m^2^	7409 (32.7)	7603 (33.6)	215 (7.45)	252 (8.73)	1972 (28.2)	2116 (30.2)
	30–35 kg/m^2^	9047 (40.0)	9090 (40.2)	377 (13.0)	362 (12.5)	2892 (41.3)	2921 (41.8)
	35–40 kg/m^2^	6479 (28.6)	6298 (27.8)	1064 (36.8)	1044 (36.1)	2220 (31.7)	2098 (30.0)
	40–45 kg/m^2^	3565 (15.7)	3418 (15.1)	1149 (39.8)	1161 (40.2)	1149 (16.4)	1136 (16.2)
	45–50 kg/m^2^	1668 (7.4)	1630 (7.2)	799 (27.6)	837 (29.0)	584 (8.4)	515 (7.4)
	50–55 kg/m^2^	762 (3.4)	715 (3.2)	464 (16.0)	488 (16.9)	231 (3.3)	215 (3.1)
	>55 kg/m^2^	454 (2.0)	436 (1.9)	357 (12.3)	372 (12.8)	152 (2.2)	137 (2.0)
HbA1c, %		8.0 (1.9)	8.1 (1.9)	6.8 (1.3)	7.2 (1.6)	7.7 (1.8)	7.9 (1.8)
HbA1c categories, *n* (%)	<6.5%	5890 (26.0)	5899 (26.0)	1540 (53.3)	1532 (53.1)	2339 (33.4)	2283 (32.6)
	6.5%–7.5%	10 194 (45.0)	10 173 (45.0)	1206 (41.8)	1220 (42.2)	3304 (47.2)	3320 (47.5)
	7.5%–8.5%	7772 (34.3)	7697 (34.0)	605 (20.9)	605 (20.9)	2010 (28.7)	2041 (29.2)
	8.5%–9.5%	4483 (19.8)	4472 (19.7)	264 (9.2)	277 (9.6)	1101 (15.7)	1101 (15.7)
	9.5%–10.5%	2519 (11.1)	2475 (10.9)	139 (4.8)	131 (4.5)	620 (8.8)	638 (9.1)
	10.5%–11.5%	1590 (7.0)	1563 (6.9)	71 (2.5)	79 (2.7)	391 (5.6)	383 (5.5)
	>11.5%	1921 (8.5)	1913 (8.5)	55 (1.9)	64 (2.2)	477 (6.8)	506 (7.2)
SBP >160 mmHg, *n* (%)		5603 (24.7)	5577 (24.6)	869 (30.1)	909 (31.5)	1620 (23.1)	1654 (23.6)
DBP >100 mmHg, *n* (%)		2277 (10.0)	2246 (9.9)	457 (15.8)	493 (17.0)	746 (10.6)	746 (10.6)
Past medical history, *n* (%)	Hypertensive diseases	17 902 (79.1)	17 946 (79.3)	2375 (82.3)	2415 (83.7)	5459 (78.1)	5417 (77.5)
	Ischaemic heart diseases	3502 (15.4)	3512 (15.5)	408 (14.1)	425 (14.7)	979 (14.0)	1014 (14.5)
	T2DM with neurological complications	3156 (13.9)	3162 (13.9)	283 (9.80)	299 (10.3)	932 (13.3)	956 (13.6)
	T2DM with kidney complications	3180 (14.0)	3208 (14.1)	218 (7.55)	235 (8.14)	883 (12.6)	916 (13.1)
	Cerebrovascular diseases	771 (3.4)	828 (3.7)	56 (1.9)	71 (2.5)	206 (2.9)	221 (3.2)
	Nicotine dependence	1868 (8.3)	1892 (8.4)	241 (8.4)	238 (8.3)	595 (8.5)	581 (8.3)
	Personal history of nicotine dependence	2643 (11.6)	2700 (11.9)	624 (21.6)	615 (21.3)	840 (12.0)	811 (11.6)
	T2DM with ophthalmic complications	994 (4.4)	982 (4.3)	76 (2.6)	76 (2.6)	306 (4.4)	314 (4.5)
	Peripheral vascular disease, unspecified	774 (3.4)	768 (3.4)	48 (1.7)	51 (1.8)	226 (3.2)	227 (3.2)
	Atherosclerosis	758 (3.4)	759 (3.4)	42 (1.5)	49 (1.7)	255 (3.7)	252 (3.6)
	Alcohol-related disorders	297 (1.3)	297 (1.3)	44 (1.5)	41 (1.4)	104 (1.5)	110 (1.6)
Persons with potential health hazards related to socioeconomic and psychosocial circumstances, *n* (%)		553 (2.4)	520 (2.3)	66 (2.3)	70 (2.4)	234 (3.3)	241 (3.4)
Medications, *n* (%)	Any lipid-lowering therapy	14 430 (63.8)	14 413 (63.7)	1279 (44.3)	1330 (46.1)	4366 (62.4)	4369 (62.5)
	Any antihypertensive therapy	2140 (9.5)	2192 (9.7)	772 (26.7)	801 (27.7)	644 (9.2)	685 (9.8)
	ACEi	6491 (28.7)	6527 (28.8)	778 (26.9)	784 (27.1)	1736 (24.8)	1734 (24.8)
	Angiotensin II inhibitors	6314 (27.9)	6333 (28.0)	600 (20.7)	612 (21.2)	2043 (29.2)	2049 (29.3)
	Insulin	6930 (30.6)	6946 (30.7)	1526 (52.8)	1563 (54.1)	1865 (26.6)	1937 (27.7)
	Metformin	13 137 (58.1)	13 146 (58.1)	1157 (40.1)	1166 (40.4)	3608 (51.6)	3651 (52.2)
	Sulfonylurea	5142 (22.7)	5241 (23.1)	328 (11.3)	357 (12.3)	1139 (16.3)	1177 (16.8)
	SGLT2i	4689 (20.7)	4630 (20.4)	117 (4.05)	121 (4.19)	1529 (21.8)	1621 (23.2)

Data are presented as number (%) for categorical variables and as median (SD) for continuous variables.

SBP, systolic blood pressure; DBP, diastolic blood pressure; ACEi: angiotensin-converting enzyme inhibitor; SGLT2i: sodium-glucose cotransporter 2 inhibitor

Supplementary data, Table S7 shows before and after matching data.

In the matched semaglutide vs DPP4i cohorts, the median follow-up was 958 [interquartile range (IQR) 819] and 882 (IQR 1058) days (∼2.5 years), respectively; corresponding follow-ups were 1535 (IQR 2054) and 1869 (IQR 2194) days (∼4.7 years) for BS vs DPP4i, and 500 (IQR 526) and 417 (IQR 598) days (∼1.3 years) for tirzepatide vs DPP4i (Supplementary data, Table S6).

### Renal outcomes

**Figure 1:**
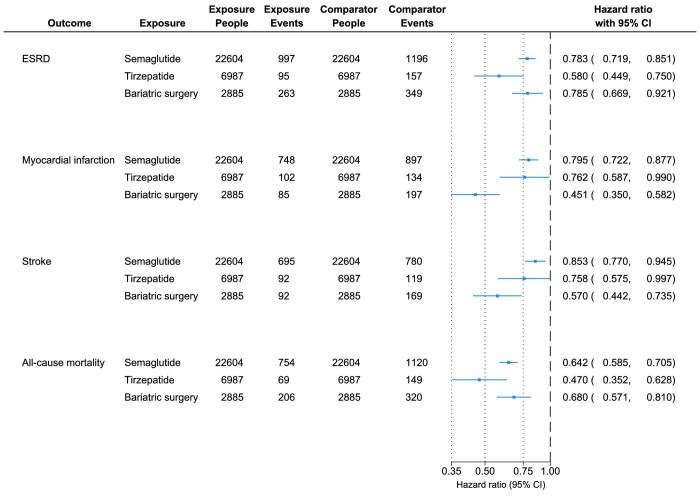
HRs of cardiorenal outcomes in people with semaglutide, BS, tirzepatide vs DPP4i in people with T2DM and CKD. ESRD was defined as starting dialysis or reaching an eGFR <15 mL/min/1.73 m^2^.

Compared with DPP4i, semaglutide showed significant renal benefits, with a 22% reduction in risk of ESRD [defined as starting dialysis or reaching an eGFR <15 mL/min/1.73 m^2^; 0.78 (0.71 to 0.85)], while tirzepatide reduced the risk of ESRD by 42% (0.58; 0.45 to 0.75) (Fig. 1). Individuals undergoing BS also had a 21% lower event rate of ESRD (0.79; 0.67 to 0.92).

### Cardiovascular disease

Compared with DPP4i, semaglutide was associated with a reduced MI risk of 20% (0.80; 0.72 to 0.88). Those using semaglutide also saw a significant 15% reduction in stroke risk (0.85; 0.77 to 0.95) (Fig. 1). Individuals treated with tirzepatide had a 24% lower event rate of MI (0.76; 0.59 to 0.99) and a 24% reduction in stroke risk (0.76; 0.58 to 1.00). BS also reduced MI risk by 55% (0.45; 0.35 to 0.58) and stroke by 43% (0.57; 0.44 to 0.74).

### All-cause mortality

The use of semaglutide was associated with a 36% significantly lower risk of all-cause mortality compared with those treated with DPP4i (0.64; 0.59 to 0.70) (Fig. 1). Tirzepatide reduced the risk of all-cause mortality by 53% (0.47; 0.35 to 0.63). BS was associated with a 32% lower risk of all-cause mortality (0.68; 0.57 to 0.81).

### Subgroup analysis

#### Sex

When stratified by sex (Supplementary data, Table S8), semaglutide significantly reduced ESRD events in both sexes [0.81 (0.72 to 0.92) in males, 0.76 (0.67 to 0.85) in females], and mortality [0.77 (0.67 to 0.87), 0.55 (0.48–0.63)]. MI and stroke benefits were observed only in females [0.72 (0.63 to 0.83), 0.65 (0.45 to 0.93)]. Tirzepatide showed significant effects only in females across all outcomes, with the lowest event rates for on mortality [0.36 (0.24 to 0.54)]. ESRD, MI and stroke were also reduced (0.58, 0.65, 0.66). BS significantly reduced MI and stroke in both sexes [e.g. MI: 0.52 (0.34 to 0.79), 0.43 (0.31 to 0.58)], and mortality in females [0.50 (0.40 to 0.63)].

#### Body mass index

Semaglutide significantly reduced all-cause mortality across both BMI groups (Supplementary data, Table S8), with larger effects in those with BMI >30 kg/m² [0.65 (0.58 to 0.73), 35%] compared with a BMI 25–30 kg/m² [0.70 (0.58 to 0.86), 30%], and also lowered ESRD, MI and stroke risks in the higher BMI group. Tirzepatide showed significant benefits only in the BMI >30 kg/m² group, reducing ESRD (44%), MI (46%) and mortality (35%) events. BS was effective in the BMI >30 kg/m² group, reducing ESRD, MI, stroke and mortality [0.48 (0.39 to 0.59), 52%], but was associated with increased mortality in the BMI 25–30 kg/m² group [2.38 (1.50–3.77)].

#### Renal function

When stratified by renal function (Supplementary data, Table S9), semaglutide significantly reduced ESRD in patients with an eGFR <45 [0.79 (0.63 to 0.99), 21%] and >60 [0.68 (0.56 to 0.83) mL/min/1.73 m^2^, 32%]. MI was reduced only in the >60 mL/min/1.73 m^2^ group [0.77 (0.66 to 0.90), 23%], while mortality was significantly lower in both eGFR 45–60 [0.63 (0.46 to 0.84), 37%] and >60 [0.70 (0.59 to 0.81), 30%] mL/min/1.73 m^2^ groups. Tirzepatide showed significantly lower rates for ESRD in the <45 mL/min/1.73 m^2^ group [0.53 (0.30 to 0.93), 47%], and stroke [0.54 (0.35 to 0.84), 46%] and mortality [0.59 (0.37 to 0.94), 41%] in the >60 mL/min/1.73 m^2^ group. BS significantly reduced the risk of ESRD in eGFR 45–60 [0.49 (0.25 to 0.96), 51%] and >60 [0.64 (0.44 to 0.92), 36%] mL/min/1.73 m^2^. MI [0.48 (0.33 to 0.68), 52%], stroke [0.55 (0.37 to 0.79), 45%] and mortality [0.75 (0.57 to 0.98), 25%] were significantly reduced in the >60 mL/min/1.73 m^2^ group, with a stronger mortality effect in the 45–60 mL/min/1.73m^2^ group [0.38 (0.19 to 0.76), 62%].

### Sensitivity analysis

#### Follow-up time

In those with at least 1 year of follow-up time (Supplementary data, Table S10), semaglutide, tirzepatide and BS were each associated with significant reductions in cardiorenal outcomes compared with DPP4i. Semaglutide reduced ESRD, MI, stroke and mortality by 18%–24%. Tirzepatide showed 31%–35% reductions in ESRD, MI and stroke, though its mortality benefit was not statistically significant. BS had the strongest effects, reducing MI and stroke by over 47%, ESRD by 28% and mortality by 38%. Compared with all participants, these reductions showed slightly more modest but consistent benefits: semaglutide reduced ESRD by 18% (vs 22% overall), tirzepatide by 31% (vs 42%–73%) and BS by 28% (vs 21%–23%), with similar patterns seen for MI, stroke and mortality.

## DISCUSSION

### Summary of findings

Here, we provide the first large-scale, real-world data on the efficacy of different weight loss interventions on mortality and cardiorenal outcomes in people with T2DM and CKD. Compared with DPP4i, semaglutide was associated with a reduced number of ESRD events. BS was also associated with reductions in ESRD, as was the use of tirzepatide. Compared with DPP4i, all interventions reduced the risk of CVD outcomes (MI and stroke), with BS and tirzepatide conferring the largest benefits. All interventions were associated with a reduced number of events for all-cause mortality, with the largest reduction following tirzepatide. Whilst the mechanism(s) of action is unclear, these data suggest that weight-lowering interventions, particularly tirzepatide, offer significant mortality and cardiorenal benefits in those with T2DM and CKD.

### Interpretation of findings

Overweight and obesity are increasingly associated with CKD and T2DM [33]. Whilst there are various intervention options for treating both body weight (loss) and concomitant disease progression, an ideal therapy would reduce body weight and directly affect pathways involved in glucose metabolism and kidney disease pathology. Whilst BS offers potent weight loss and possible renoprotective mechanisms, access to BS is challenging, particularly in scale, and it may not be acceptable to all. Classes of GLP1-RA-based therapies, such as semaglutide and tirzepatide, may offer the triple effects desired, including having direct kidney protective effects [15] and a significant impact on glucose lowering, weight loss [34] and other cardiometabolic risk factors, such as blood pressure [35, 36].

#### Renal outcomes

Semaglutide potentiates glucose-dependent secretion of insulin, suppresses glucagon secretion, slows gastric emptying and promotes satiety [37]. The largest RCT to date testing the effects of semaglutide in 3533 people with CKD and T2DM, the FLOW trial, was terminated early after semaglutide reduced the risk of kidney outcomes and death from cardiovascular causes [18]. A secondary analysis of the SELECT trial reported a 22% reduction in composite kidney endpoint following semaglutide use in over 8800 patients with obesity (without diabetes), and this was driven by a decrease in the onset of persistent macroalbuminuria and ≥50% reduction in eGFR; however, the effects of semaglutide on delaying the initiation of KRT were unclear due to a low number of events [17]. A recent meta-analysis of 11 GLP1-RA RCTs in T2DM, including FLOW and SELECT, showed a 16% reduction in the risk of kidney failure compared with placebo [15]. With RCTs not powered to separately detect effects on rates of long-term outcomes such as kidney failure, there remains a paucity of data from high-quality and large, real-world studies in people living with T2DM and CKD [15].

Our data suggest that, compared with DPP4i, a superior effect on renal outcomes was seen following tirzepatide use, with a 42% reduction in the risk of ESRD. This is larger than that seen following semaglutide use (22%). There was no noticeable difference in the rates of ESRD between semaglutide and BS vs DPP4i. However, the cohort characteristics varied across analyses, potentially limiting the validity of directly comparing the different exposed groups. The mechanism underpinning these effects on kidney outcomes is unclear. Weight loss likely contributes to some degree, and weight loss from BS in people with obesity has been shown to have renoprotective mechanisms, including a reduction in glomerular hyperfiltration, proteinuria, cardiometabolic parameters and adipose-related inflammation [8, 38]. The SELECT trial suggested that 81% of the change in eGFR at Week 104 could be mediated by body weight change [17]. Yet, in the FLOW trial, the effect of semaglutide was unrelated to changes in body weight, suggesting that semaglutide may offer independent mechanisms of kidney protection; these are likely multifactorial and, based on experimental models and biomarker data, may include improved blood pressure, and decreases in glucose, inflammation, oxidative stress and fibrosis [34]. Intrinsic kidney and immune cells contain the GLP-1 receptor, and GLP1-RAs could reduce cellular expression of proinflammatory and profibrotic mediators [34, 39, 40]. Along with greater weight loss, the superior kidney-protective effects of tirzepatide may be attributed to the dual activation of GIP and GLP-1 receptors exerting direct beneficial effects on kidney tissues, potentially reducing inflammation and fibrosis [41, 42]. Whilst participants were matched for BMI in our analysis, we do not have data concerning the change in body weight, so we cannot draw a conclusion about its relative contribution to outcome events.

#### Cardiovascular outcomes

Semaglutide has been shown to have robust cardiovascular protective effects. In people with T2DM and CKD, the FLOW trial found the risk of major cardiovascular events (defined as a composite of nonfatal MI, nonfatal stroke or death from cardiovascular causes) was 18% (HR 0.82; 95% CI 0.68 to 0.98) lower in the semaglutide group [18]. In adults with overweight/obesity, without T2DM and generally no CKD (SELECT trial), semaglutide reduced the risk of cardiovascular events by ∼20% (HR 0.80; 95% CI 0.72 to 0.90) [16], and in the SUSTAIN-6 trial, semaglutide reduced non-fatal MI in those with T2DM (a third of who had an eGFR <60 mL/min/1.73 m^2^) by 26% and non-fatal stroke by 39% [43]. In our data, semaglutide was associated with a similar reduction in cardiovascular-related outcomes to the ∼20% lower event rates reported in contemporary clinical trials [16, 18, 43] (15% in stroke and 20% in MI).

In our data, tirzepatide showed robust effects in reducing CVD-related outcomes (24% in both stroke and in MI). In the SUMMIT trial, tirzepatide reduced the combined risk of CVD-related death and worsening heart failure events by 38% compared with placebo in adults with heart failure with preserved ejection fraction, and obesity [44]. Whilst the SURPASS-CVOT trial, powered to investigate the risk of major adverse cardiovascular events (MACE) with tirzepatide in individuals with obesity, is ongoing [45], the efficacy of tirzepatide in reducing CVD remains unclear. However, a meta-analysis encompassing seven RCTs from the SURPASS program assessed tirzepatide’s cardiovascular safety in people with T2DM. Tirzepatide had a relatively neutral effect on CVD-outcomes and did not increase the risk of MACE compared with control treatments, with an HR of 0.80 (0.57 to 1.11). Specifically, the HR for non-fatal MI was 0.80 (0.51 to 1.25), and stroke was 0.81 (0.39, 1.68).

The largest reductions in CVD-related outcomes were seen following BS, which reduced the risk of MI (55%) and stroke (43%). However, it should be noted that our BS population were of higher BMI and a younger age than the other cohorts. The effects of BS on cardiovascular outcomes are well-described in previous studies, with BS associated with large reductions in the risk of CVD and cardiovascular events such as atherosclerosis and ischaemic heart disease [46]. These effects are likely mediated, in part, by weight loss and improvement in CVD risk factors such as atherosclerosis, hypertension and hyperlipidaemia, ventricular hypertrophy regression, improved diastolic function [47] and better glycaemic control [8].

#### All-cause mortality

Semaglutide was associated with a reduction in all-cause mortality (by 36%) compared with DPP4i. Clinical trial data support the beneficial effect of semaglutide on mortality [15]. For example, death from any cause was reduced by 19% (0.81; 0.71 to 0.93) in SELECT (obesity without diabetes and mostly without CKD) and by 20% (0.80; 0.67 to 0.95) in the FLOW trial (CKD and diabetes) [18]. However, no difference in all-cause mortality was seen between semaglutide and placebo in the SUSTAIN-6 trial (T2DM) [43]. Whilst tirzepatide reduced all-cause mortality vs DPP4i by 53%, this was observed over a relatively short follow-up period with a low number of events and must be interpreted cautiously. Previous TriNetX research, including a retrospective analysis involving 140 308 patients with T2DM, where tirzepatide, compared with those receiving GLP1-RAs, was associated with a 42% lower risk of all-cause mortality [20]. However, clinical trial data are unclear. In those without T2DM, a meta-analysis of the SURMOUNT RCTs found tirzepatide did not reduce all-cause mortality (0.69; 0.24 to 1.95) [21]. Similar results have been reported in a meta-analysis of the SURPASS trials (0.80; 0.51 to 1.25) [48], whilst, interestingly, the decreased rate of all-cause mortality for tirzepatide versus DPP4i remains larger than that reported in clinical trials, where no statistically significant mortality benefit has been observed. This discrepancy likely reflects residual confounding and selection bias inherent to observational data, despite propensity-score matching. Additionally, limitations in death reporting within the TriNetX platform may affect mortality ascertainment [49, 50]. Although conceivably, the effects on mortality may be partly mediated by the protective effect of GLP1-RA-based therapies on other bodily systems as described above, these factors, combined with the shorter follow-up duration and lower event rates in the tirzepatide cohort, necessitate cautious interpretation of the mortality finding, and further work is needed to confirm the protective effect on mortality.

Whilst it is difficult to infer from our findings, interestingly, dual activation of GIP/GLP-1 receptors may provide additional benefits over the activation of GLP-1 receptors alone. From a renal perspective, studies suggest that GIP may attenuate local adipose tissue inflammation and reduce proinflammatory cytokine levels [51], which may act favourably on the pathogenesis of diabetic kidney disease. GLP-1 receptors are present on preglomerular vascular smooth muscle, glomerular and tubular cells and, thus, may directly affect the kidney [52] via inhibition of glomerular hyperfiltration or proximal tubular diuresis [52, 53]. Cardiovascular-wise, tirzepatide has a superior effect on lipid profile, inflammation, blood pressure and atherosclerosis, and with GIP receptors expressed in cardiac and vascular tissues, their activation may improve endothelial function, reduce inflammation and enhance myocardial metabolism [20, 54, 55].

Analyses stratified by sex, BMI, renal function and follow-up duration revealed generally consistent patterns of benefit across interventions, though with some variation. Semaglutide significantly reduced ESRD and mortality across sexes and BMI categories, aligning with meta-analyses showing consistent benefits in both men and women [56]. Tirzepatide’s effects were more pronounced in females and individuals with BMI >30 kg/m², consistent with emerging evidence suggesting enhanced cardiovascular and renal benefits in higher BMI strata [57]. BS demonstrated strong cardiovascular and renal benefits, particularly in those with preserved renal function and higher BMI, though it was associated with increased mortality in the lower BMI subgroup, an observation supported by population-based studies [58]. Importantly, when restricted to individuals with ≥1 year of follow-up, effect sizes for all interventions were attenuated, suggesting that shorter follow-up may have inflated initial estimates. This phenomenon is well documented in survival analysis literature, where HRs can be sensitive to follow-up duration, especially when the proportional hazards assumption is violated [59]. These findings underscore the importance of cautious interpretation, particularly given potential residual confounding and the observational nature of the data. While the subgroup results support the overall conclusions, they highlight the need for longer-term and more granular data to confirm differential effects across patient subpopulations.

### Strengths and limitations

Our study has many strengths. TriNetX offers unprecedented access to large-scale, real-world patient data and enables rapid cohort identification across multiple healthcare organizations [50]. Our study includes a large sample, comparisons across a range of glucose-lowering interventions and extensive propensity-score matching (in particular, using objective laboratory and anthropometric values such as HbA1c and BMI). We chose DPP4i as our comparator, given that it is a common glucose-lowering therapy and it has a neutral effect on body weight [60]. As recommended by recent contemporary cardio-renal literature [61], we defined a composite of ESRD as either the first episode of dialysis or defined as an eGFR <15 mL/min/1.73 m^2^.

However, we also acknowledge several limitations. Interpretation of data, particularly around tirzepatide use, is limited in some cases by short follow-up times (<∼1 year) and a low number of events. The shorter follow-up time in the tirzepatide cohort is likely driven by its recent availability. Although sensitivity analyses in patients with >1 year of follow-up showed consistent benefits, the reduced duration may limit the robustness of long-term outcome estimates, particularly for mortality. Additionally, differences in cohort characteristics and follow-up durations mean that direct comparisons between treatment groups should be interpreted cautiously. While truncating follow-up to a common timeframe could reduce bias, it would also restrict the ability to observe longer-term effects, particularly for interventions like BS. Instead, we conducted analyses restricted to individuals with ≥1 year of follow-up, which showed consistent but more modest benefits across outcomes. These findings support the validity of our results while reflecting the evolving nature of real-world data.

As with other datasets utilizing electronic health records, TriNETx may be prone to errors in coding accuracy and variable completeness across contributing healthcare organizations. While the platform harmonizes data centrally, certain fields, such as the death recording [50], medication adherence and longitudinal laboratory values may be incomplete or delayed in recording, which could affect outcome ascertainment. We could not identify individual medication adherence or the duration of exposure, as these data are unavailable. Concerning semaglutide, we could not differentiate between oral vs subcutaneous formulations and doses of semaglutide/tirzepatide, as these data are not available. As discussed, residual and unmeasured confounding remains possible, particularly around socioeconomic determinants of health and lifestyle factors. Most individuals across each cohort had relatively mild CKD status, and thus, many may be unlikely to have reached our kidney-related endpoints during the follow-up time. It is important to note that the BS cohort differed substantially in baseline characteristics, including being ∼10 years younger and predominantly female (74%), which reflects real-world patterns in surgical eligibility and uptake. While propensity-score matching was used to balance clinical variables between the BS and DPP4i groups, residual differences may persist and could influence observed outcomes. Therefore, as already stated, comparisons across intervention groups should be interpreted cautiously. The multiple comparisons across intervention groups may increase the risk of type I error, and our statistically significant findings warrant caution due to the possibility of ‘false-positive’ results.

## CONCLUSION

The management of overweight and obesity is an important cornerstone of the clinical management of people with T2DM and CKD. Here, using a large real-world dataset, we show that compared with DPP4i, semaglutide, tirzepatide and BS—interventions shown to elicit weight loss and improve glycaemic control—have many benefits. Whilst inappropriate to directly compare weight loss interventions to each other, the largest reductions in risk were seen with tirzepatide vs DPP4i for renal, cardiovascular and mortality outcomes. Our data suggest that all investigated weight-lowering interventions are effective in reducing cardiorenal outcomes in individuals with T2DM, CKD and overweight/obesity; this supports the use of tirzepatide and semaglutide as non-surgical interventions in real-world populations for patients with T2DM and CKD.

## Supplementary Material

gfaf258_Supplemental_File

## Data Availability

The TriNetX US Collaborative Network returned the results of these analyses as ‘.csv’ files, which we downloaded and archived. Aggregate data, as presented in this paper, were acquired from TriNetX. This study had no special privileges. Eligibility criteria specified in the Methods and Supplementary Material would allow other researchers to identify similar cohorts of patients as used for our analyses; however, TriNetX is a live platform with new data being added daily so exact counts will vary. To gain access to the data, a request can be made to TriNetX (join@trinetx.com), but costs might be incurred, and a data-sharing agreement would be necessary.
