# Supplementary material for: Cardiorenal outcomes of weight loss interventions in people with CKD and type 2 diabetes
Source: Nephrol Dial Transplant. 2025 Dec 4;41(7):1262–73. doi: 10.1093/ndt/gfaf258 (PMC13314376; doi:10.1093/ndt/gfaf258)
Supplement: gfaf258_Supplemental_File [file gfaf258_supplemental_file.docx]

**Supplementary material**

**Supplementary Table 1.** Reporting of studies Conducted using Observational Routinely-collected health Data (RECORD) checklist

**Supplementary Table 2.** Procedure codes used to identify people undergoing bariatric surgery

**Supplementary Table 3**. Clinical codes used to identify people with previous renal pathology

# **Supplementary Table 4.** Clinical codes used to define outcomes

# **Supplementary Table 5.** Clinical codes used for diagnoses used in propensity score matching

# **Supplementary Table 6.** Follow-up time after matching

# **Supplementary Table 7.** Baseline characteristics of the cohorts before and after propensity score matching

**Supplementary Table 8.** Hazard ratios of cardiorenal outcomes in people with semaglutide, bariatric surgery, insulin vs DPP4i in people with type 2 diabetes and chronic kidney disease, stratified by sex and BMI

**Supplementary Table 9.** Hazard ratios of cardiorenal outcomes in people with semaglutide, bariatric surgery, insulin vs DPP4i in people with type 2 diabetes and chronic kidney disease, stratified by renal function

**Supplementary Table 10.** Hazard ratios of cardiorenal outcomes in people with semaglutide, bariatric surgery, insulin vs DPP4i in people with type 2 diabetes and chronic kidney disease, in those with >1 year of follow-up

# **Supplementary Figure 1.** Kaplan-Meier event-free probability over time

**Supplementary Table 1.** Reporting of studies Conducted using Observational Routinely-collected health Data (RECORD) checklist

|  | **Item No.** | **STROBE items** | **Location (page) in manuscript where items are reported** | **RECORD items** | **Location (page) in manuscript where items are reported** |
| --- | --- | --- | --- | --- | --- |
| Title and abstract | | | | | |
|  | 1 | (a) Indicate the study’s design with a commonly used term in the title or the abstract (b) Provide in the abstract an informative and balanced summary of what was done and what was found | 1, 3 | RECORD 1.1: The type of data used should be specified in the title or abstract. When possible, the name of the databases used should be included.  RECORD 1.2: If applicable, the geographic region and timeframe within which the study took place should be reported in the title or abstract.  RECORD 1.3: If linkage between databases was conducted for the study, this should be clearly stated in the title or abstract. | 1, 3 |
| Introduction | | | | | |
| Background rationale | 2 | Explain the scientific background and rationale for the investigation being reported | 4, 5 |  |  |
| Objectives | 3 | State specific objectives, including any prespecified hypotheses | 5 |  |  |
| Methods | | | | | |
| Study Design | 4 | Present key elements of study design early in the paper | 6 |  |  |
| Setting | 5 | Describe the setting, locations, and relevant dates, including periods of recruitment, exposure, follow-up, and data collection | 6, 7 |  |  |
| Participants | 6 | *(a) Cohort study* - Give the eligibility criteria, and the sources and methods of selection of participants. Describe methods of follow-up  *Case-control study* - Give the eligibility criteria, and the sources and methods of case ascertainment and control selection. Give the rationale for the choice of cases and controls  *Cross-sectional study* - Give the eligibility criteria, and the sources and methods of selection of participants  *(b) Cohort study* - For matched studies, give matching criteria and number of exposed and unexposed  *Case-control study* - For matched studies, give matching criteria and the number of controls per case | 6 | RECORD 6.1: The methods of study population selection (such as codes or algorithms used to identify subjects) should be listed in detail. If this is not possible, an explanation should be provided.  RECORD 6.2: Any validation studies of the codes or algorithms used to select the population should be referenced. If validation was conducted for this study and not published elsewhere, detailed methods and results should be provided.  RECORD 6.3: If the study involved linkage of databases, consider use of a flow diagram or other graphical display to demonstrate the data linkage process, including the number of individuals with linked data at each stage. | 6 |
| Variables | 7 | Clearly define all outcomes, exposures, predictors, potential confounders, and effect modifiers. Give diagnostic criteria, if applicable. | 6, 7 | RECORD 7.1: A complete list of codes and algorithms used to classify exposures, outcomes, confounders, and effect modifiers should be provided. If these cannot be reported, an explanation should be provided. | Supp material |
| Data sources/ measurement | 8 | For each variable of interest, give sources of data and details of methods of assessment (measurement).  Describe comparability of assessment methods if there is more than one group | N/A |  |  |
| Bias | 9 | Describe any efforts to address potential sources of bias | 7 |  |  |
| Study size | 10 | Explain how the study size was arrived at | 7 |  |  |
| Quantitative variables | 11 | Explain how quantitative variables were handled in the analyses. If applicable, describe which groupings were chosen, and why | 7 |  |  |
| Statistical methods | 12 | (a) Describe all statistical methods, including those used to control for confounding  (b) Describe any methods used to examine subgroups and interactions  (c) Explain how missing data were addressed  (d) *Cohort study* - If applicable, explain how loss to follow-up was addressed  *Case-control study* - If applicable, explain how matching of cases and controls was addressed  *Cross-sectional study* - If applicable, describe analytical methods taking account of sampling strategy  (e) Describe any sensitivity analyses | 7 |  |  |
| Data access and cleaning methods |  | .. |  | RECORD 12.1: Authors should describe the extent to which the investigators had access to the database population used to create the study population.  RECORD 12.2: Authors should provide information on the data cleaning methods used in the study. | 6, 7 |
| Linkage |  | .. |  | RECORD 12.3: State whether the study included person-level, institutional-level, or other data linkage across two or more databases. The methods of linkage and methods of linkage quality evaluation should be provided. |  |
| Results | | | | | |
| Participants | 13 | (a) Report the numbers of individuals at each stage of the study (*e.g.*, numbers potentially eligible, examined for eligibility, confirmed eligible, included in the study, completing follow-up, and analysed)  (b) Give reasons for non-participation at each stage.  (c) Consider use of a flow diagram | 9 | RECORD 13.1: Describe in detail the selection of the persons included in the study (*i.e.,* study population selection) including filtering based on data quality, data availability and linkage. The selection of included persons can be described in the text and/or by means of the study flow diagram. | 9 |
| Descriptive data | 14 | (a) Give characteristics of study participants (*e.g.*, demographic, clinical, social) and information on exposures and potential confounders  (b) Indicate the number of participants with missing data for each variable of interest  (c) *Cohort study* - summarise follow-up time (*e.g.*, average and total amount) | 9, Table |  |  |
| Outcome data | 15 | *Cohort study* - Report numbers of outcome events or summary measures over time  *Case-control study* - Report numbers in each exposure category, or summary measures of exposure  *Cross-sectional study* - Report numbers of outcome events or summary measures | 9 |  |  |
| Main results | 16 | (a) Give unadjusted estimates and, if applicable, confounder-adjusted estimates and their precision (e.g., 95% confidence interval). Make clear which confounders were adjusted for and why they were included  (b) Report category boundaries when continuous variables were categorized  (c) If relevant, consider translating estimates of relative risk into absolute risk for a meaningful time period | 9 |  |  |
| Other analyses | 17 | Report other analyses done—e.g., analyses of subgroups and interactions, and sensitivity analyses | 9 |  |  |
| Discussion | | | | | |
| Key results | 18 | Summarise key results with reference to study objectives | 12 |  |  |
| Limitations | 19 | Discuss limitations of the study, taking into account sources of potential bias or imprecision. Discuss both direction and magnitude of any potential bias | 14, 15 | RECORD 19.1: Discuss the implications of using data that were not created or collected to answer the specific research question(s). Include discussion of misclassification bias, unmeasured confounding, missing data, and changing eligibility over time, as they pertain to the study being reported. | 14, 15 |
| Interpretation | 20 | Give a cautious overall interpretation of results considering objectives, limitations, multiplicity of analyses, results from similar studies, and other relevant evidence | 15 |  |  |
| Generalisability | 21 | Discuss the generalisability (external validity) of the study results | 14, 15 |  |  |
| Other Information | | | | | |
| Funding | 22 | Give the source of funding and the role of the funders for the present study and, if applicable, for the original study on which the present article is based | 7, 8 |  |  |
| Accessibility of protocol, raw data, and programming code |  | .. |  | RECORD 22.1: Authors should provide information on how to access any supplemental information such as the study protocol, raw data, or programming code. | Supp material |

*Reference: Benchimol EI, Smeeth L, Guttmann A, Harron K, Moher D, Petersen I, Sørensen HT, von Elm E, Langan SM, the RECORD Working Committee. The REporting of studies Conducted using Observational Routinely-collected health Data (RECORD) Statement. *PLoS Medicine* 2015; in press.

*Checklist is protected under Creative Commons Attribution ([CC BY](http://creativecommons.org/licenses/by/4.0/)) license.

**Supplementary Table 2.** Procedure codes used to identify people undergoing bariatric surgery

| **Coding system** | **Reference** | **Code name** |
| --- | --- | --- |
| **CPT** | 43644 | Laparoscopy, surgical, gastric restrictive procedure; with gastric bypass and Roux-en-Y gastroenterostomy (roux limb 150 cm or less) |
|  | 43645 | Laparoscopy, surgical, gastric restrictive procedure; with gastric bypass and small intestine reconstruction to limit absorption |
|  | 43770 | Laparoscopy, surgical, gastric restrictive procedure; placement of adjustable gastric restrictive device (eg, gastric band and subcutaneous port components) |
|  | 43775 | Laparoscopy, surgical, gastric restrictive procedure; longitudinal gastrectomy (ie, sleeve gastrectomy) |
|  | 43842 | Gastric restrictive procedure, without gastric bypass, for morbid obesity; vertical-banded gastroplasty |
|  | 43843 | Gastric restrictive procedure, without gastric bypass, for morbid obesity; other than vertical-banded gastroplasty |
|  | 43845 | Gastric restrictive procedure with partial gastrectomy, pylorus-preserving duodenoileostomy and ileoileostomy (50 to 100 cm common channel) to limit absorption (biliopancreatic diversion with duodenal switch) |
|  | 43846 | Gastric restrictive procedure, with gastric bypass for morbid obesity; with short limb (150 cm or less) Roux-en-Y gastroenterostomy |
|  | 43847 | Gastric restrictive procedure, with gastric bypass for morbid obesity; with small intestine reconstruction to limit absorption |
|  | 1007403 | Gastric restrictive procedure, without gastric bypass, for morbid obesity |
|  | 1014146 | Gastric restrictive procedure, with gastric bypass for morbid obesity |
|  | 1014146 | Gastric restrictive procedure, with gastric bypass for morbid obesity |
| **ICD-10-PCS** | 0D1607A | Bypass Stomach to Jejunum with Autologous Tissue Substitute, Open Approach |
|  | 0D160JA | Bypass Stomach to Jejunum with Synthetic Substitute, Open Approach |
|  | 0D160KA | Bypass Stomach to Jejunum with Nonautologous Tissue Substitute, Open Approach |
|  | 0D160ZA | Bypass Stomach to Jejunum, Open Approach |
|  | 0D1687A | Bypass Stomach to Jejunum with Autologous Tissue Substitute, Via Natural or Artificial Opening Endoscopic |
|  | 0D168JA | Bypass Stomach to Jejunum with Synthetic Substitute, Via Natural or Artificial Opening Endoscopic |
|  | 0D168KA | Bypass Stomach to Jejunum with Nonautologous Tissue Substitute, Via Natural or Artificial Opening Endoscopic |
|  | 0D168ZA | Bypass Stomach to Jejunum, Via Natural or Artificial Opening Endoscopic |
|  | 0DV64CZ | Restriction of Stomach with Extraluminal Device, Percutaneous Endoscopic Approach |
| **SNOMED CT** | 30803004 | Printen and Mason operation, high gastric bypass |
|  | 173747005 | Roux-en-Y gastrojejunostomy |
|  | 426738005 | Duodenal switch |
|  | 427074001 | Laparoscopic sleeve gastrectomy |
|  | 427980007 | Sleeve gastrectomy with duodenal switch |

**CPT:** Current Procedural Terminology; **ICD-10-PCD:** International Classification of Diseases Procedure Coding System (10^th^ edition); **SNOMED CT:** Systematized Nomenclature of Medicine Clinical Terms

# **Supplementary Table 3**. Clinical codes used to identify people with previous renal pathology

| **Coding system** | **Reference** | **Code name** |
| --- | --- | --- |
| **CPT** | Z94.0 | Kidney transplant status |
| **ICD-9-CM** | 39.95 | Hemodialysis |
| **ICD-10-CM** | 1012740 | Dialysis Services and Procedures |
|  | C64 | Malignant neoplasm of kidney, except renal pelvis |
|  | C65 | Malignant neoplasm of renal pelvis |
|  | C66 | Malignant neoplasm of ureter |
|  | N00-N08 | Glomerular diseases |
|  | N10-N16 | Renal tubulo-interstitial diseases |
|  | N17 | Acute kidney failure |
|  | N18.6 | End stage renal disease |
|  | N19 | Unspecified kidney failure |
|  | N25-N29 | Other disorders of kidney and ureter |
|  | Q61.1 | Polycystic kidney, infantile type |
|  | Q61.2 | Polycystic kidney, adult type |
|  | Q61.3 | Polycystic kidney, unspecified |
|  | Z99.2 | Dependence on renal dialysis |
| **SNOMED CT** | 70536003 | Transplant of kidney |
|  | 108241001 | Dialysis procedure |
|  | 175902000 | Cadaveric renal transplant |
|  | 302497006 | Hemodialysis |

**CPT:** Current Procedural Terminology; **ICD-9-CM:** International Classification of Diseases Clinical Modification (9^th^ revision); **ICD-10-CM:** International Classification of Diseases Clinical Modification (10^th^ revision); **SNOMED CT:** Systematized Nomenclature of Medicine Clinical Terms

# **Supplementary Table 4.** Clinical codes used to define outcomes

| **Outcome** | **Coding system** | **Reference** | **Code name** |
| --- | --- | --- | --- |
| **Dialysis** | **CPT** | 90935 | Hemodialysis procedure with single evaluation by a physician or other qualified health care professional |
|  |  | 90937 | Hemodialysis procedure requiring repeated  evaluation(s) with or without substantial  revision of dialysis prescription |
|  |  | 90999 | Unlisted dialysis procedure, inpatient or  outpatient |
|  |  | 1012757 | Miscellaneous Dialysis Services and  Procedures |
|  | **ICD-9-CM** | 39.95 | Hemodialysis |
|  | **ICD-10-CM** | Z99.2 | Dependence on renal dialysis |
|  | **SNOMED CT** | 108241001 | Dialysis procedure |
|  |  | 302497006 | Hemodialysis |
| **Transplant** | **CPT** | 1008109 | Renal allotransplantation, implantation of graft |
|  | **SNOMED CT** | 70536003 | Transplant of kidney |
|  | **SNOMED CT** | 175902000 | Cadaveric renal transplant |
| **Myocardial infarction** | **ICD-10-CM** | I21 | Acute myocardial infarction |
| **Stroke** | **ICD-10-CM** | I63 | Cerebral infarction |
|  | **ICD-10-CM** | I61 | Nontraumatic intracerebral hemorrhage |

**CPT:** Current Procedural Terminology; **ICD-9-CM:** International Classification of Diseases Clinical Modification (9^th^ revision); **ICD-10-CM:** International Classification of Diseases Clinical Modification (10^th^ revision); **SNOMED CT:** Systematized Nomenclature of Medicine Clinical Terms

# **Supplementary Table 5.** Clinical codes used for diagnoses used in propensity score matching

| **Coding system** | **Reference** | **Code name** |
| --- | --- | --- |
| **ICD-10-CM** | E11.2 | Type 2 diabetes mellitus with kidney complications |
|  | E11.3 | Type 2 diabetes mellitus with ophthalmic complications |
|  | E11.4 | Type 2 diabetes mellitus with neurological complications |
|  | F10 | Alcohol related disorders |
|  | F17 | Nicotine dependence |
|  | I10-I1A | Hypertensive diseases |
|  | I20-I25 | Ischemic heart diseases |
|  | I60-I69 | Cerebrovascular diseases |
|  | I70 | Atherosclerosis |
|  | I73.9 | Peripheral vascular disease, unspecified |
|  | Z55- | Persons with potential health hazards related to |
|  | Z65 | socioeconomic and psychosocial circumstances |

**ICD-10-CM:** International Classification of Diseases Clinical Modification (10^th^ revision)

# **Supplementary Table 6.** Follow-up time after matching

| **Analysis** | **Cohort** | **Mean follow-up time (days)** | **Standard deviation** | **Median follow-up time (days)** | **Interquartile range** |
| --- | --- | --- | --- | --- | --- |
| Semaglutide vs. DPP4i | Semaglutide | 1022 | 581 | 958 | 819 |
|  | DPP4i | 973 | 704 | 882 | 1058 |
| Bariatric surgery vs. DPP4i | Bariatric surgery | 1886 | 1478 | 1535 | 2054 |
|  | DPP4i | 2031 | 1446 | 1869 | 2194 |
| Tirzepatide vs. DPP4i | Tirzepatide | 502 | 307 | 500 | 526 |
|  | DPP4i | 503 | 414 | 417 | 598 |

**DPP4i:** Dipeptidyl peptidase-4 inhibitor

# **Supplementary Table** **7.** Baseline characteristics of the cohorts before and after propensity score matching

|  | | **Semaglutide v. DPP4i** | | | | **Bariatric surgery v. DPP4i** | | | | **Tirzepatide v. DPP4i** | | | |
| --- | --- | --- | --- | --- | --- | --- | --- | --- | --- | --- | --- | --- | --- |
|  |  | **Before matching** | | **After matching** | | **Before matching** | | **After matching** | | **Before matching** | | **After matching** | |
|  |  | **Exposure** | **Comparator (DPP4i)** | **Exposure** | **Comparator (DPP4i)** | **Exposure** | **Comparator (DPP4i)** | **Exposure** | **Comparator (DPP4i)** | **Exposure** | **Comparator (DPP4i)** | **Exposure** | **Comparator (DPP4i)** |
| Number | | 61,046 | 64,222 | 22,604 | 22,604 | 4,572 | 6,2653 | 2,885 | 2,885 | 16,969 | 62,509 | 6,987 | 6,987 |
| Age at Index | | 61.4 (10.9) | 64.5 (10.6) | 63.9 (10.2) | 64.3 (11.5) | 53.2 (10.6) | 64.5 (10.6) | 56.1 (9.96) | 56.0 (12.2) | 60.7 (10.7) | 64.5 (10.6) | 63.4 (9.88) | 63.9 (11.6) |
| Current Age | | 63.9 (10.8) | 71.6 (10.6) | 67.3 (10.0) | 67.7 (11.0) | 59.8 (11.6) | 71.6 (10.6) | 63.3 (10.8) | 63.4 (12.5) | 62.0 (10.6) | 71.6 (10.6) | 65.1 (9.71) | 65.6 (11.3) |
| Female | | 37,781 (61.8) | 35,057 (54.5) | 13,019 (57.5) | 12,962 (57.3) | 3,605 (78.8) | 34,203 (54.5) | 2,146 (74.3) | 2,136 (74.0) | 10,484 (61.7) | 34,080 (54.5) | 4,075 (58.3) | 4,057 (58.0) |
| Race | American Indian or Alaska Native | 412 (0.7) | 299 (0.5) | 155 (0.7) | 134 (0.6) | 29 (0.6) | 298 (0.5) | 17 (0.6) | 15 (0.5) | 92 (0.5) | 299 (0.5) | 52 (0.7) | 37 (0.5) |
|  | Asian | 1,862 (3.1) | 2,961 (4.6) | 913 (4.0) | 951 (4.2) | 47 (1.0) | 2,935 (4.7) | 37 (1.3) | 40 (1.4) | 350 (2.1) | 2,934 (4.7) | 205 (2.9) | 207 (3.0) |
|  | Black or African American | 8,847 (14.4) | 11,206 (17.4) | 3,688 (16.3) | 3,738 (16.5) | 880 (19.2) | 11,079 (17.6) | 530 (18.3) | 556 (19.2) | 1,856 (10.9) | 10,856 (17.3) | 911 (13.0) | 924 (13.2) |
|  | Native Hawaiian or Other Pacific Islander | 468 (0.8) | 591 (0.9) | 190 (0.8) | 175 (0.8) | 16 (0.4) | 591 (0.9) | 16 (0.6) | 16 (0.6) | 108 (0.6) | 592 (0.9) | 54 (0.8) | 44 (0.6) |
|  | White | 1691 (2.8) | 2,195 (3.4) | 772 (3.4) | 805 (3.6) | 163 (3.6) | 2,117 (3.7) | 101 (3.5) | 100 (3.5) | 410 (2.4) | 2,104 (3.4) | 222 (3.2) | 252 (3.6) |
|  | Other Race | 45,238 (74.1) | 43,340 (67.4) | 15,781 (69.8) | 15,677 (69.3) | 3150 (68.8) | 42,014 (67.0) | 2020 (70.0) | 1,979 (68.5) | 13453 (79.2) | 42,109 (67.3) | 5,223 (74.7) | 5,170 (73.9) |
|  | Unknown Race | 2,528 (4.1) | 3,630 (5.6) | 1105 (4.9) | 1,124 (5.0) | 287 (6.3) | 3,619 (5.8) | 164 (5.7) | 179 (6.2) | 700 (4.1) | 3,615 (5.8) | 320 (4.6) | 353 (5.1) |
| eGFR mL/min/1.73m2 | | 65.6 (16.0) | 64.8 (19.2) | 64.3 (16.5) | 65.6 (18.7) | 70.0 (18.0) | 64.8 (19.3) | 69.1 (18.8) | 70.6 (21.8) | 65.6 (15.5) | 64.8 (19.3) | 64.0 (16.4) | 65.7 (17.9) |
| eGFR categories | 65-75 mL/min/1.73m2 | 40,026 (65.5) | 39,066 (60.8) | 14,164 (62.6) | 14,123 (62.4) | 3,298 (72.1) | 38,129 (60.8) | 1,995 (69.1) | 2,013 (69.7) | 11,369 (66.9) | 37,950 (60.7) | 4474 (64.0) | 4,457 (63.7) |
|  | 55-65 mL/min/1.73m2 | 25,648 (42.0) | 29,294 (45.6) | 9,951 (44.0) | 9,946 (44.0) | 2,000 (43.7) | 28,634 (45.7) | 1,321 (45.7) | 1,332 (46.1) | 6,951 (40.9) | 28,554 (45.6) | 2,949 (42.2) | 2,930 (41.9) |
|  | 45-55 mL/min/1.73m^2^ | 12,937 (21.1) | 18,776 (29.2) | 5,810 (25.7) | 5,839 (25.8) | 918 (20.0) | 18,373 (29.3) | 664 (23.0) | 695 (24.0) | 3178 (18.7) | 18,318 (29.3) | 1533 (21.9) | 1,573 (22.5) |
|  | 35-45 mL/min/1.73m^2^ | 5,471 (9.0) | 9,720 (15.1) | 2,780 (12.2) | 2,828 (12.5) | 333 (7.28) | 9,529 (15.2) | 277 (9.60) | 293 (10.1) | 1252 (7.37) | 9,506 (15.2) | 701 (10.0) | 724 (10.3) |
|  | 25-35 mL/min/1.73m^2^ | 1862 (3.1) | 3,965 (6.2) | 1019 (4.5) | 1,039 (4.6) | 88 (1.9) | 3,889 (6.2) | 78 (2.7) | 77 (2.7) | 420 (2.5) | 3,880 (6.2) | 262 (3.8) | 283 (4.5) |
|  | 15-25 mL/min/1.73m^2^ | 436 (0.7) | 1120 (1.7) | 262 (1.2) | 280 (1.2) | 20 (0.4) | 1104 (1.8) | 18 (0.6) | 20 (0.7) | 111 (0.7) | 1103 (1.8) | 72 (1.0) | 89 (1.3) |
|  | <15 mL/min/1.73m^2^ | 1,930 (3.2) | 3,154 (4.9) | 792 (3.5) | 791 (3.5) | 280 (6.1) | 3142 (5.0) | 173 (6.0) | 172 (60) | 283 (1.7) | 3,137 (5.0) | 133 (1.9) | 126 (1.8) |
| HbA1c (%) | | 7.7 (1.8) | 8.1 (1.8) | 8.0 (1.9) | 8.1 (1.9) | 6.6 (1.2) | 8.1 (1.8) | 6.8 (1.3) | 7.2 (1.6) | 7.3 (1.7) | 8.1 (1.8) | 7.7 (1.8) | 7.9 (1.8) |
| HbA1c categories | <6.5 % | 24,213 (39.6) | 15,054 (23.4) | 5,890 (26.0) | 5,899 (26.0) | 2,928 (64.0) | 14,648 (23.3) | 1540 (53.3) | 1,532 (53.1) | 8,141 (47.9) | 14,595 (23.3) | 2,339 (33.4) | 2,283 (32.6) |
|  | 6.5-7.5 % | 27,776 (45.5) | 29,531 (45.9) | 10,194 (45.0) | 10,173 (45.0) | 1,595 (34.8) | 28,706 (45.8) | 1206 (41.8) | 1220 (42.2) | 7739 (45.6) | 28,678 (45.8) | 3304 (47.2) | 3,320 (47.5) |
|  | 7.5-8.5 % | 16,412 (26.8) | 23,780 (37.0) | 7,772 (34.3) | 7,697 (34.0) | 730 (15.9) | 23,069 (36.8) | 605 (20.9) | 605 (20.9) | 3495 (20.5) | 23,059 (36.8) | 2010 (28.7) | 2,041 (29.2) |
|  | 8.5-9.5 % | 9,245 (15.1) | 13,338 (20.7) | 4,483 (19.8) | 4,472 (19.7) | 304 (6.64) | 12,978 (20.7) | 264 (9.15) | 277 (9.6) | 1845 (10.8) | 12,966 (20.7) | 1101 (15.7) | 1,101 (15.7) |
|  | 9.5-10.5 % | 5,395 (8.83) | 7,083 (11.0) | 2,519 (11.1) | 2,475 (10.9) | 159 (3.5) | 6,905 (11.0) | 139 (4.81) | 131 (4.5) | 1131 (6.7) | 6,873 (10.9) | 620 (8.87) | 638 (9.13) |
|  | 10.5-11.5 % | 3,569 (5.84) | 4,114 (6.40) | 1,590 (7.03) | 1,563 (6.9) | 81 (1.8) | 4,033 (6.4) | 71 (2.5) | 79 (2.7) | 717 (4.2) | 4,024 (6.4) | 391 (5.59) | 383 (5.5) |
|  | >11.5% | 4,229 (6.92) | 4,759 (7.41) | 1,921 (8.49) | 1,913 (8.46) | 57 (1.24) | 4,672 (7.5) | 55 (1.9) | 64 (2.2) | 940 (5.5) | 4,654 (7.4) | 477 (6.82) | 506 (7.2) |
| BMI | | 36.7 (7.45) | 33.5 (6.75) | 34.8 (7.08) | 34.2 (6.96) | 44.0 (8.50) | 33.5 (6.8) | 42.4 (8.65) | 42.0 (8.6) | 37.5 (7.5) | 33.5 (6.8) | 35.5 (7.01) | 34.4 (6.9) |
| BMI | 0-25 kg/m2 | 1733 (2.83) | 4,156 (6.47) | 1113 (4.92) | 1,190 (5.26) | 130 (2.84) | 4043 (6.5) | 112 (3.88) | 129 (4.5) | 305 (1.8) | 4072 (6.5) | 238 (3.40) | 254 (3.6) |
|  | 25-30 kg/m2 | 13160 (21.5) | 24,865 (38.7) | 7409 (32.7) | 7,603 (33.6) | 219 (4.79) | 24,286 (38.7) | 215 (7.45) | 252 (8.7) | 2944 (17.3) | 24,383 (39.0) | 1972 (28.2) | 2,116 (30.2) |
|  | 30-35 kg/m2 | 22974 (37.6) | 26,075 (40.6) | 9047 (40.0) | 9,090 (40.2) | 412 (9.01) | 25,439 (40.6) | 377 (13.0) | 362 (12.5) | 6117 (36.0) | 25,513 (40.8) | 2892 (41.3) | 2,921 (41.8) |
|  | 35-40 kg/m2 | 20777 (34.0) | 16,413 (25.5) | 6479 (28.6) | 6,298 (27.8) | 1461 (31.9) | 16,017 (25.5) | 1,064 (36.8) | 1,044 (36.1) | 6175 (36.3) | 16,073 (25.7) | 2,220 (31.7) | 2,098 (30.0) |
|  | 40-45 kg/m2 | 13306 (21.7) | 8,361 (13.0) | 3565 (15.7) | 3,418 (15.1) | 1919 (41.9) | 8,144 (12.9) | 1,149 (39.8) | 1,161 (40.2) | 4055 (23.8) | 8,158 (13.0) | 1,149 (16.4) | 1,136 (16.2) |
|  | 45-50 kg/m2 | 6956 (11.3) | 3,848 (6.0) | 1668 (7.4) | 1,630 (7.2) | 1570 (34.3) | 3,741 (6.0) | 799 (27.6) | 837 (29.0) | 2094 (12.3) | 3,750 (6.0) | 584 (8.35) | 515 (7.4) |
|  | 50-55 kg/m2 | 3252 (5.32) | 1,631 (2.54) | 762 (3.37) | 715 (3.16) | 1045 (22.8) | 1,595 (2.5) | 464 (16.0) | 488 (16.9) | 1043 (6.14) | 1,608 (2.6) | 231 (3.30) | 215 (3.1) |
|  | At least 55 kg/m2 | 2,018 (3.30) | 1,035 (1.61) | 454 (2.00) | 436 (1.92) | 830 (18.1) | 1,017 (1.62) | 357 (12.3) | 372 (12.8) | 647 (3.81) | 1,017 (1.6) | 152 (2.17) | 137 (2.0) |
| SBP >160 mmHg | | 13,967 (22.8) | 16,887 (26.2) | 5,603 (24.7) | 5,577 (24.6) | 1373 (30.0) | 16,506 (26.3) | 869 (30.1) | 909 (31.5) | 3,597 (21.1) | 16,453 (26.3) | 1,620 (23.1) | 1,654 (23.6) |
| DBP >100 mmHg | | 6,638 (10.8) | 6,315 (9.8) | 2,277 (10.0) | 2,246 (10.0) | 803 (17.5) | 6,210 (9.9) | 457 (15.8) | 493 (17.0) | 1,846 (10.8) | 6,233 (10.0) | 746 (10.6) | 746 (10.6) |
| Past medical history | Hypertensive diseases | 46,733 (76.5) | 51,509 (80.2) | 17,902 (79.1) | 17,946 (79.3) | 3765 (82.3) | 50,305 (80.2) | 2375 (82.3) | 2,415 (83.7) | 12,573 (74.0) | 50,196 (80.3) | 5,459 (78.1) | 5,417 (77.5) |
|  | Ischemic heart diseases | 8,408 (13.7) | 10,708 (16.6) | 3,502 (15.4) | 3,512 (15.5) | 563 (12.3) | 10,485 (16.7) | 408 (14.1) | 425 (14.7) | 2,049 (12.0) | 10,466 (16.7) | 979 (14.0) | 1,014 (14.5) |
|  | Type 2 diabetes mellitus with kidney complications | 7,187 (11.7) | 7,670 (11.9) | 3,180 (14.0) | 3,208 (14.1) | 291 (6.36) | 7,625 (12.1) | 218 (7.55) | 235 (8.14) | 1,656 (9.75) | 7,612 (12.1) | 883 (12.6) | 916 (13.1) |
|  | Type 2 diabetes mellitus with neurological complications | 7,108 (11.6) | 7,521 (11.7) | 3,156 (13.9) | 3,162 (13.9) | 403 (8.81) | 7,496 (11.9) | 283 (9.80) | 299 (10.3) | 1783 (10.5) | 7,501 (12%) | 932 (13.3) | 956 (13.6) |
|  | Personal history of nicotine dependence | 7,347 (12.0) | 6,161 (9.59) | 2,643 (11.6) | 2,700 (11.9) | 1268 (27.7) | 6,099 (9.7) | 624 (21.6) | 615 (21.3) | 2093 (12.3) | 6,085 (9.7) | 840 (12.0) | 811 (11.6) |
|  | Nicotine dependence | 4,911 (8.0) | 4,786 (7.5) | 1,868 (8.3) | 1,892 (8.4) | 378 (8.3) | 4,677 (7.5) | 241 (8.35) | 238 (8.25) | 1281 (7.5) | 4,656 (7.4) | 595 (8.5) | 581 (8.3) |
|  | Cerebrovascular diseases | 1,692 (2.8) | 2,517 (3.91) | 771 (3.41) | 828 (3.7) | 65 (1.4) | 2,444 (3.9) | 56 (1.9) | 71 (2.46) | 404 (2.4) | 2,431 (3.9) | 206 (2.9) | 221 (3.2) |
|  | Type 2 diabetes mellitus with ophthalmic complications | 2,320 (3.8) | 2,541 (4.0) | 994 (4.4) | 982 (4.3) | 100 (2.18) | 2,497 (4.0) | 76 (2.6) | 76 (2.63) | 545 (3.2) | 2,489 (4.0) | 306 (4.4) | 314 (4.5) |
|  | Atherosclerosis | 1,745 (2.9) | 1,929 (3.0) | 758 (3.4) | 759 (3.4) | 53 (1.2) | 1,894 (3.0) | 42 (1.5) | 49 (1.7) | 470 (2.8) | 1,884 (3.0) | 255 (3.7) | 252 (3.6) |
|  | Peripheral vascular disease, unspecified | 1,711 (2.8) | 2,214 (3.4) | 774 (3.4) | 768 (3.4) | 62 (1.4) | 2,173 (3.5) | 48 (1.7) | 51 (1.8) | 431 (2.5) | 2,176 (3.5) | 226 (3.2) | 227 (3.2) |
| Persons with potential health hazards related to socioeconomic and psychosocial circumstances | | 1,449 (2.4) | 1,019 (1.6) | 553 (2.44) | 520 (2.3) | 125 (2.7) | 1,004 (1.6) | 66 (2.3) | 70 (2.4) | 523 (3.1) | 999 (1.6) | 234 (3.3) | 241 (3.4) |
| Alcohol related disorders |  | 820 (1.3) | 796 (1.2) | 297 (1.3) | 297 (1.3) | 73 (1.6) | 783 (1.3) | 44 (1.5) | 41 (1.4) | 263 (1.6) | 780 (1.2) | 104 (1.5) | 110 (1.6) |
| Medications | Any lipid lowering therapy | 35,857 (58.7) | 42,028 (65.4) | 14,430 (63.8) | 14,413 (63.7) | 1,538 (33.6) | 41,120 (65.6) | 1279 (44.3) | 1,330 (46.1) | 9,361 (55.1) | 41,038 (65.6) | 4366 (62.4) | 4,369 (62.5) |
|  | Any antihypertensive therapy | 4,576 (7.5) | 7,426 (11.5) | 2,140 (9.5) | 2,192 (9.7) | 1,549 (33.8) | 7,382 (11.7) | 772 (26.7) | 801 (27.7) | 1,348 (7.9) | 7,358 (11.7) | 644 (9.2) | 685 (9.8) |
|  | ACEi | 15,369 (25.1) | 22,397 (34.8) | 6,491 (28.7) | 6,527 (28.8) | 1,005 (21.9) | 21,903 (34.9) | 778 (26.9) | 784 (27.1) | 3,606 (21.2) | 21,842 (34.9) | 1736 (24.8) | 1,734 (24.8) |
|  | Angiotensin II inhibitors | 16,608 (27.2) | 16,986 (26.4) | 6,314 (27.9) | 6,333 (28.0) | 791 (17.3) | 16,670 (26.6) | 600 (20.7) | 612 (21.2) | 4,731 (27.8) | 16,620 (26.5) | 2043 (29.2) | 2,049 (29.3) |
|  | Insulin | 14,066 (23.0) | 20,719 (32.2) | 6,930 (30.6) | 6,946 (30.7) | 2,407 (52.6) | 20,435 (32.6) | 1,526 (52.8) | 1,563 (54.1) | 3,249 (19.1) | 20,417 (32.6) | 1865 (26.6) | 1,937 (27.7) |
|  | Metformin | 30,593 (50.1) | 40,530 (63.1) | 13,137 (58.1) | 13,146 (58.1) | 1,449 (31.6) | 39,594 (63.1) | 1,157 (40.1) | 1,166 (40.4) | 7,153 (42.1) | 39,500 (63.1) | 3608 (51.6) | 3,651 (52.2) |
|  | Sulfonylureas | 9,316 (15.2) | 20,652 (32.1) | 5,142 (22.7) | 5,241 (23.1) | 357 (7.80) | 20,181 (32.2) | 328 (11.3) | 357 (12.3) | 1,764 (10.3) | 20,161 (32.2) | 1139 (16.3) | 1,177 (16.8) |
|  | SGLT2i | 10,702 (17.5) | 7,460 (11.6) | 4,689 (20.7) | 4,630 (20.4) | 134 (2.93) | 7,316 (11.6) | 117 (4.05) | 121 (4.19) | 2,618 (15.4) | 7,320 (11.7) | 1529 (21.8) | 1,621 (23.2) |

Data are presented as number (%) for categorical variables and as median (SD) for continuous variables.

**DPP4i:** Dipeptidyl peptidase-4 inhibitor; **eGFR:** Estimated glomerular filtration rate**; BMI:** Body mass index; **SBP:** Systolic blood pressure; **DBP:** Diastolic blood pressure; **ACEi:** Angiotensin-converting-enzyme inhibitor; **SGLT2i:** Sodium-glucose co-transporter-2 inhibitor

**Supplementary Table 8.** Hazard ratios of cardiorenal outcomes in people with semaglutide, bariatric surgery, insulin vs DPP4i in people with type 2 diabetes and chronic kidney disease, stratified by sex and BMI

|  | **Male** | | **Female** | | **BMI 25-30 kg/m2** | | **BMI >30 kg/m2** | |
| --- | --- | --- | --- | --- | --- | --- | --- | --- |
| **Semaglutide vs DPP4i** |  |  |  |  |  |  |  |  |
| ESRD | **0.81 (0.72 to 0.92)** | **19%** | **0.76 (0.67 to 0.85)** | **24%** | 0.82 (0.68 to 1.00) | 18% | **0.79 (0.71 to 0.88)** | **21%** |
| MI | 0.92 (0.80 to 1.06 | 8% | **0.72 (0.63 to 0.83)** | **28%** | 0.87 (0.70 to 1.07) | 13% | **0.76 (0.67 to 0.87)** | **24%** |
| Stroke | 0.85 (0.73 to 1.00 | 15% | **0.65 (0.45 to 0.93)** | **35%** | 0.86 (0.69 to 1.07) | 14% | **0.84 (0.74 to 0.96)** | **16%** |
| All-cause mortality | **0.77 (0.67 to 0.87** | **23%** | **0.55 (0.48 to 0.63)** | **45%** | **0.70 (0.58 to 0.86)** | **30%** | **0.65 (0.58 to 0.73)** | **35%** |
|  |  |  |  |  |  |  |  |  |
| **Tirzepatide vs DPP4i** |  |  |  |  |  |  |  |  |
| ESRD | 0.84 (0.57 to 1.24) | 16% | **0.58 (0.41 to 0.83)** | **42%** | 0.89 (0.42 to 1.91) | 11% | **0.56 (0.41 to 0.76)** | **44%** |
| MI | 1.03 (0.70 to 1.53) | +3% | **0.65 (0.45 to 0.93)** | **35%** | 1.18 (0.64 to 2.15) | +18% | **0.54 (0.38 to 0.76)** | **46%** |
| Stroke | 0.98 (0.65 to 1.49) | 2% | **0.66 (0.45 to 0.95)** | **34%** | 0.76 (0.43 to 1.32) | 24% | 0.69 (0.47 to 1.02) | 31% |
| All-cause mortality | 0.68 (0.45 to 1.03) | 32% | **0.36 (0.24 to 0.54)** | **64%** | 0.58 (0.30 to 1.11) | 42% | **0.45 (0.31 to 0.66)** | **35%** |
|  |  |  |  |  |  |  |  |  |
| **Bariatric surgery vs DPP4i** |  |  |  |  |  |  |  |  |
| ESRD | 0.79 (0.58 to 1.07) | 21% | **0.76 (0.63 to 0.92)** | **24%** | 0.65 (0.32 to 1.33) | 35% | **0.81 (0.68 to 0.96)** | **19%** |
| MI | **0.52 (0.34 to 0.79)** | **48%** | **0.43 (0.31 to 0.58)** | **37%** | Not enough cases | - | **0.46 (0.35 to 0.61)** | **54%** |
| Stroke | **0.60 (0.38 to 0.95)** | **40%** | **0.54 (0.40 to 0.72)** | **46%** | Not enough cases | - | **0.57 (0.43 to 0.75)** | **43%** |
| All-cause mortality | 1.07 (0.81 to 1.41) | +7% | **0.50 (0.40 to 0.63)** | **50%** | **2.38 (1.50 to 3.77)** | **+138%** | **0.48 (0.39 to 0.59)** | **52%** |

Abbreviations: **ESRD**: End stage renal disease; **DPP4i:** Dipeptidyl peptidase-4 inhibitor; **CI:** Confidence Interval. ESRD was defined as starting dialysis or reaching an eGFR <15 mL/min/1.73m^2^

**Supplementary Table 9.** Hazard ratios of cardiorenal outcomes in people with semaglutide, bariatric surgery, insulin vs DPP4i in people with type 2 diabetes and chronic kidney disease, stratified by renal function

|  | **eGFR <45** | | **eGFR 45-60** | | **eGFR >60** | |
| --- | --- | --- | --- | --- | --- | --- |
| **Semaglutide vs DPP4i** |  |  |  |  |  |  |
| ESRD | **0.79 (0.63 to 0.99)** | **21%** | 0.94 (0.69 to 1.28) | 6% | **0.68 (0.56 to 0.83)** | **32%** |
| MI | 0.83 (0.58 to 1.18) | 17% | 0.78 (0.58 to 1.04) | 22% | **0.77 (0.66 to 0.90)** | **23%** |
| Stroke | 0.96 (0.65 to 1.40) | 4% | 0.75 (0.54 to 1.04) | 25% | 0.95 (0.82 to 1.11) | 5% |
| All-cause mortality | 0.78 (0.59 to 1.02) | 22% | **0.63 (0.46 to 0.84)** | **37%** | **0.70 (0.59 to 0.81)** | **30%** |
|  |  |  |  |  |  |  |
| **Tirzepatide vs DPP4i** |  |  |  |  |  |  |
| ESRD | **0.53 (0.30 to 0.93)** | **47%** | Not enough cases | - | 0.98 (0.60 to 1.60) | 2% |
| MI | Not enough cases | - | Not enough cases | - | 0.75 (0.51 to 1.10) | 25% |
| Stroke | Not enough cases | - | 1.06 (0.52 to 2.13) | +6% | **0.54 (0.35 to 0.84)** | **46%** |
| All-cause mortality | Not enough cases | - | 0.77 (0.35 to 1.71) | 23% | **0.59 (0.37 to 0.94)** | **41%** |
|  |  |  |  |  |  |  |
| **Bariatric surgery vs DPP4i** |  |  |  |  |  |  |
| ESRD | Not enough cases | - | **0.49 (0.25 to 0.96)** | **51%** | **0.64 (0.44 to 0.92)** | **36%** |
| MI | Not enough cases | - | Not enough cases | - | **0.48 (0.33 to 0.68)** | **52%** |
| Stroke | Not enough cases | - | Not enough cases | - | **0.55 (0.37 to 0.79)** | **45%** |
| All-cause mortality | Not enough cases | **-** | **0.38 (0.19 to 0.76)** | **62%** | **0.75 (0.57 to 0.98)** | **25%** |

Abbreviations: **ESRD**: End stage renal disease; **DPP4i:** Dipeptidyl peptidase-4 inhibitor; **CI:** Confidence Interval. ESRD was defined as starting dialysis or reaching an eGFR <15 mL/min/1.73m^2^

**Supplementary Table 10.** Hazard ratios of cardiorenal outcomes in people with semaglutide, bariatric surgery, insulin vs DPP4i in people with type 2 diabetes and chronic kidney disease, in those with >1 year of follow up

| **Semaglutide vs DPP4i** |  |  |
| --- | --- | --- |
| ESRD | 0.82 (0.75 to 0.90) | 18% |
| MI | 0.81 (0.73 to 0.90) | 19% |
| Stroke | 0.80 (0.71 to 0.89) | 20% |
| All-cause mortality | 0.76 (0.68 to 0.85) | 24% |
|  |  |  |
| **Tirzepatide vs DPP4i** |  |  |
| ESRD | 0.69 (0.52 to 0.91) | 31% |
| MI | 0.66 (0.49 to 0.91) | 34% |
| Stroke | 0.65 (0.47 to 0.89) | 35% |
| All-cause mortality | 0.67 (0.43 to 1.03) | 33% |
|  |  |  |
| **Bariatric surgery vs DPP4i** |  |  |
| ESRD | 0.72 (0.61 to 0.86) | 28% |
| MI | 0.48 (0.38 to 0.62) | 52% |
| Stroke | 0.53 (0.42 to 0.68) | 47% |
| All-cause mortality | 0.62 (0.51 to 0.76) | 38% |

Abbreviations: **ESRD**: End stage renal disease; **DPP4i:** Dipeptidyl peptidase-4 inhibitor; **CI:** Confidence Interval. ESRD was defined as starting dialysis or reaching an eGFR <15 mL/min/1.73m^2^

# **Supplementary Figure 1.** Kaplan-Meier event-free probability over time

Dashed lines represent 95% confidence interval. Note than the scale of the y-axis varies with the relative incidence of outcomes. The x axis varies with the duration of follow-up. The unexposed group received DPP4 inhibitors.

ESRD: End-stage renal disease; BS: Bariatric surgery; MI: Myocardial infarction; ACM: All-cause mortality
